# The Application of Granulated Expanded Glass Aggregate with Cement Grout as an Alternative Solution for Sub-Grade and Frost-Protection Sub-Base Layer in Road Construction

**DOI:** 10.3390/ma12213528

**Published:** 2019-10-28

**Authors:** Marzena Kurpińska, Beata Grzyl, Marek Pszczola, Adam Kristowski

**Affiliations:** Gdansk University of Technology, Faculty of Civil and Environmental Engineering, 80-233 Gdansk, Poland; beata.grzyl@pg.edu.pl (B.G.); marek.pszczola@pg.edu.pl (M.P.); adam.kristowski@pg.edu.pl (A.K.)

**Keywords:** road design, building construction, sub-grade layer, permeable frost protection layer, lightweight aggregate, granulated ash aggregate, artificial neural networks

## Abstract

The purpose of the research was to assess the possibility of using granulated expanded glass aggregate (GEGA) with cement grout as a replacement of a sub-grade and frost-protection layer, made of natural fine aggregates (NATU), stabilized with a hydraulic binder. Instead of traditional parts of the road construction, such as the sub-grade and frost-protection layer with the application of fine aggregate, stabilized with cement, the authors propose only one layer, made of lightweight water-permeable material, containing GEGA with a grain size from 8 to 11.2 mm. In the article the authors present the physical properties of the materials, applied for the road layers, the properties of the fine aggregate, stabilized with cement, and those of the cement composite with GEGA as an alternative solution. The laboratory test results of fine aggregates, stabilized with cement and of cement composites with GEGA, are presented. Porosity, volume density, compressive strength, and frost resistance are being researched. The results of those tests are meant to play an essential role in designing the thickness of road layers. Different types of pavement structure (asphalt and concrete) and different values of road load are being considered in the given work. The paper is concluded with considerations on an innovative solution, involving the use of ecological materials.

## 1. Introduction

The road construction sector all around the world is the largest consumer of natural aggregates [1]. It is reported that global annual use of the aggregates constitutes over 40 billion tons, 90% of which is produced on the basis of natural resources. The rest are aggregates, made of the recycled materials (about 5%), synthetic aggregates (2%) and aggregates, extracted from the sea area (about 2%) [2]. Despite the presence of a number of areas, rich in natural aggregate sources, certain countries are characterized by a deficit of natural aggregates. It is often the case that a fine aggregate is inapplicable in construction because of too small a grain size.

The use of industrial by-products in road construction can contribute to the discussion on reducing the consumption of natural resources and reducing the areas of landfills. Glass waste could be considered as a potential alternative secondary raw material in road construction [3,4,5,6]. Waste glass is an excellent material to be subjected to repeated recycling [7,8]. At the same time, due to its composition and structure, glass does not pose any hazard to the environment as a recycled material. It is estimated that annually, in Europe, 11–40 kg of glass waste is generated per capita, depending on the country. Overall production of waste is constantly growing. Glass constitutes approximately 10%–15% of municipal waste, depending on the waste management practices in the country.

According to the World Bank, annual waste production is about 2 billion tons [9,10,11,12]. The development of waste glass processing technology allows reaching up to 100% recycling, which contributes to the reduction of energy consumption for processing primary raw materials for the production of glass, i.e., sand, soda, and limestone dust [13,14]. There are many concerns about the industrial use of the recycled products. With this in mind, many studies are being carried out, confirming the possibility of using waste materials, including glass cullet [15]. Expanded glass aggregate, where cullet is the key component, is proposed to be applied for the improvement of the road sub-grade. The possibility of using shredded waste glass in road engineering, as a substitute for coarse aggregate, was carried out by [16,17,18,19]. Another solution was the use of shredded glass instead of natural sand. Such studies were conducted by [20,21,22,23]. Another lightweight aggregate, which has already been used in road construction, is produced from expanded clay or fly ash, coming from coal combustion or municipal waste incineration plants. It has been well-known and successfully used for quite a long time [24,25,26,27,28,29]. The examples of application of lightweight aggregate fillers, presented in the publications, confirm that this solution is technically feasible in the road construction industry, and its significant advantages influence the possibility of reducing costs of road construction as compared to traditional materials [30,31,32,33,34,35,36,37,38,39]. Therefore, the authors have made an attempt to use GEGA as a substitute for the natural aggregate in the sub-grade and the frost protection layer of the road foundation. The main assumption, which is made here, is that construction material should meet all the requirements, related to, among others, mechanical properties, durability, and economic coefficient [40,41].

The authors put forward the thesis that it is possible to use GEGA of diameter 8/11.2 mm with cement grout, instead of two layers: a sub-grade and a frost-protection layer of the road. At the same time, special attention has been paid to the quality and the cost of the recycled materials. The main assumption was that the quality, cost, and durability of the new solution cannot turn out less beneficial for the interested parties than the solution, traditionally used in road construction. The main profit of the new solution implementation can be seen in lower consumption of natural resources, application of waste glass and, therefore, a reduction of CO_2_ emission.

On the basis of the research, carried out by [42,43], the authors assessed the suitability of the glass waste in the form of foam glass as an alternative to natural sand, used as a sub-grade and a frost-protection layer of the pavement road.

The authors of the article below undertook a research into the possibilities of using GEGA from the waste glass in road construction. The main goal of the research was to find a new solution that would allow reducing the consumption of natural resources and, instead, to use the material derived from glass waste recycling.

## 2. Materials and Methods

### 2.1. Assumptions of Pavement Structure and Adopted Materials

**Figure 1:**
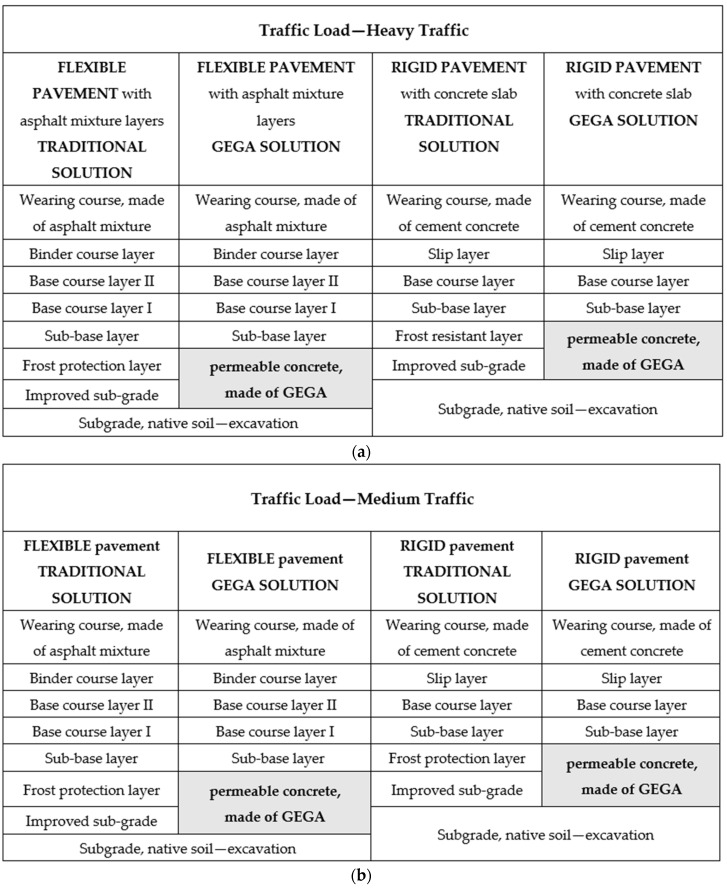

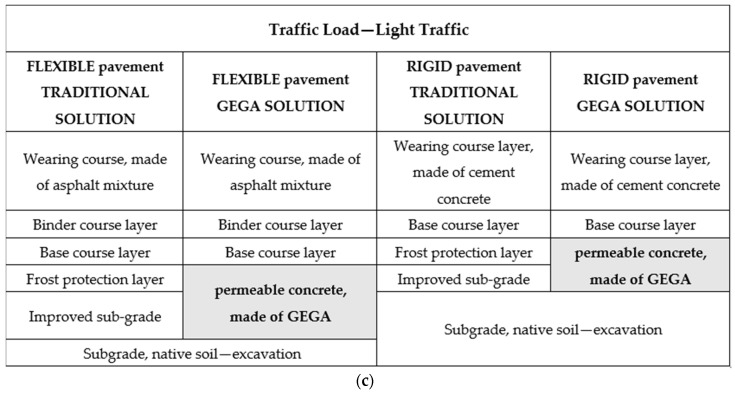
Structure of road layers design for flexible and rigid pavements—traditional and with the use of permeable concrete, made of GEGA for traffic load: (**a**) heavy traffic, (**b**) medium traffic, and (**c**) light traffic.

Two main types of pavement structure were analyzed: type 1—flexible pavement with asphalt mixture layers and base course, made of an unbound mixture, and type 2—rigid pavement with a concrete slab in a wearing course layer and base course layer, made of a mixture, bound with a hydraulic binder. Traffic categories were marked in accordance with the guidelines for the road pavement design in Poland [44]. For flexible pavements, road structures for heavy traffic (22.00–52.00 million equivalent standard axle load (ESAL) of 100 kN/lane), for medium traffic (2.50–7.30 million of ESAL of 100 kN/lane) and for light traffic (0.09–0.50 million of ESAL of 100 kN/lane) were assumed. Additionally, there were subsurface and groundwater conditions for cohesive soils adopted, e.g., sandy clay, and high groundwater level. In the analyzed examples, the proposed new solution, which was assumed, was frost protection layer performing the function of a drainage layer at the same time. For rigid pavements, road structures for heavy traffic (42.63–101.25 million of ESAL of 100 kN/lane), for medium traffic (6.39–15.99 million of ESAL of 100 kN/lane), and for light traffic (0.15–0.75 million of ESAL of 100 kN/lane) were assumed. Additionally, subsurface and groundwater conditions, as well as conditions for the application of a drainage layer, were assumed analogously as for flexible pavements. The layout of type 1 and 2 pavement layers is schematically shown in Figure 1.

The new solution assumes the substitution of the traditional solution with one, single layer, made of permeable lightweight GEGA concrete, constituting, at the same time, a frost protection course and improved soil sub-grade. The characteristic feature of the permeable lightweight GEGA concrete is liquid permeability. Porous lightweight GEGA concrete is freeze and thaw resistant. Permeable concretes can be made of a single- or double- fraction aggregate with a grain size of more than 4 mm. The amount of cement paste shall be used in the quantity, allowing to cover individual grains of the aggregate and to create an interfacial transition zone (ITZ) between the grains of the aggregate.

Figure 1 shows structural layers of flexible and rigid pavements, made of traditional and permeable concrete, and the ones, made of permeable concrete with solutions using GEGA for traffic load: heavy, medium, and light traffic.

Apparent, the density of condensed fine aggregate, stabilized with cement, ranges up to 1700–2000 kg/m^3^ whereas, in the case of using artificial aggregates with the addition of fly ash or clay, the apparent density reaches only 1400 kg/m^3^. If we use GEGA, the apparent density does not exceed 1000 kg/m^3^. The size of the spaces between the grains can be controlled to some extent by the grain size of the coarse aggregate. The amount and the size of the spaces between the grains depends on the diameter of the aggregate and on the amount of cement grout surrounding the grains of the aggregate and partially filling the pores. The greater the void content of the aggregate composition is, and the less pore filling is, the larger the porosity and the higher the water permeability are. Additionally, the amount of slurry or mortar will influence the quality of ITZ between the grains of the aggregate and cement grout. With the increase of the amount of slurry and mortar, mechanical properties of the composite will be more beneficial. The overall porosity of the composite and, therefore, permeability change with the thickness of the ITZ. The higher the amount of slurry is, the thicker the contact zone is and, at the same time, the higher compression strength is.

### 2.2. Materials

Portland cement CEM II/A-V 42.5 N with 20% fly ash, according to EN 197-1, was used to perform the tests. Chemical content and physical properties of the cement CEM II/A-V 42.5 N are shown in Table 1. The tests have been carried out in the laboratory of Gdańsk University of Technology.

**Figure 2:**
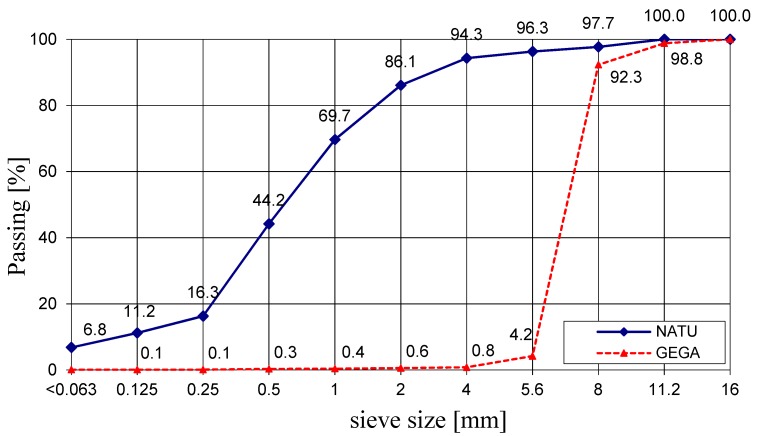
Distribution curves of aggregates size.

**Figure 3:**
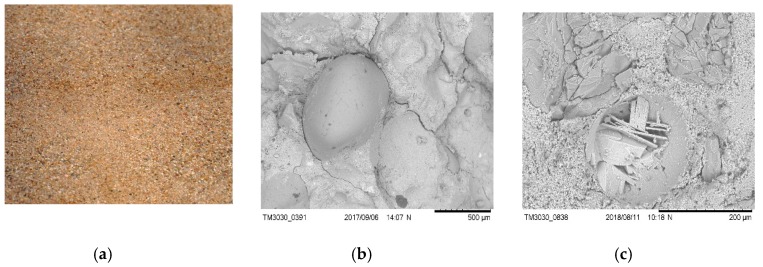
Aggregate NATU (**a**) grains to 4 mm; (**b**) grain surface, 100×; (**c**) grain structure after cutting the sample (grain in the vicinity of cement paste), 1000×.

The main component of cement is CaO, and its content is 54%, while the content of SiO_2_ silica is approximately 25%. Natural fine aggregate with a grain size from 0 to 4 mm (NATU), meeting the requirements of EN 12620: 2010, was used for the test. Distribution curves of NATU are shown in Figure 2. The chemical composition of NATU is shown in Table 2 and Table 3. Figure 3 presents microscopic images of fine aggregate grains in the vicinity of cement paste.

**Figure 4:**
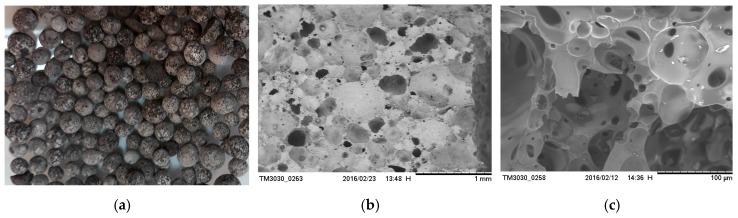
Granulated expanded glass aggregate GEGA (**a**); grains at 11.2 mm; (**b**) structure of GEGA, 200×; (**c**) structure of GEGA, 1000×.

Artificial aggregate (GEGA) is manufactured from clear construction glass recycling and municipal waste recycling. The resources for clear glass production are quartz sand and additives, such as: sodium and calcium carbonate, flux: boron and lead oxide. Glass waste is ground in a ball mill and, next, cement is added, as well as fly ash, zeolite, metakaolin, foaming substances, and water. Then, out of the mix the granules are formed and placed in the furnace at 900 °C. Finally, a light-gray or beige porous granulated product is obtained. Granulated aggregate is sorted according to grain diameter. For this research GEGA (Figure 4) with a grain size ranging from 8 to 11.2 mm was applied. Its chemical and physical properties are shown in Table 2 and Table 3.

The main component of NATU and GEGA is SiO_2_ silica, and its content in NATU is 97.5% and in GEGA is 63.3%.

### 2.3. Preparation of Mix and Samples

**Figure 5:**
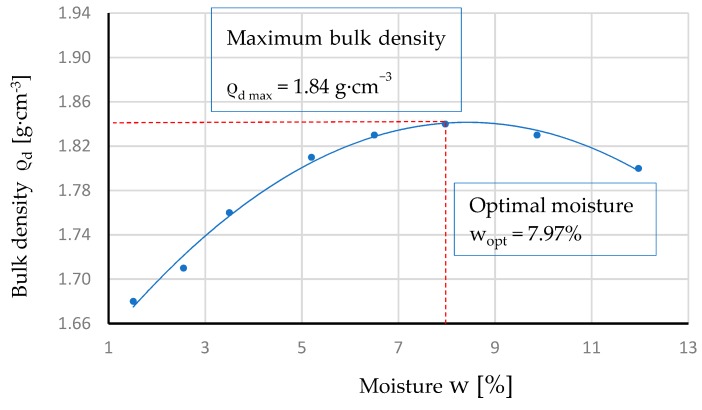
Relationship of the bulk density of NATU *ρ_d_* with moisture w.

Research of the properties was carried out for two variants of the material, marked NATU and GEGA. In the first variant, NATU, the samples were representative for the material, used in the cement-stabilized sub-grade and for a frost protection road layer in Poland. A mixture of NATU, stabilized with cement, contained 20% fly ash. Properties of the components are presented in Table 1, Table 2 and Table 3. NATU mixture, bound with a hydraulic binder, was prepared with CEM II/A-V 42.5 N cement, 129 kg; NATU, 1742 kg; and water, 107 kg. The composition was designed on the basis of the tests, aiming to determine the maximum density and optimal moisture content of the material. The components of the mixture were mixed in a mechanical mixer. First, NATU was dry-mixed with cement for 2 min, then water was added and mixed for another 3 min. The maximum density of NATU with cement and water of max = 1.84 g/cm^3^ was determined and the optimum water content constituted up to 7.97%. The relationship of bulk density and moisture is shown in Figure 5.

**Figure 6:**
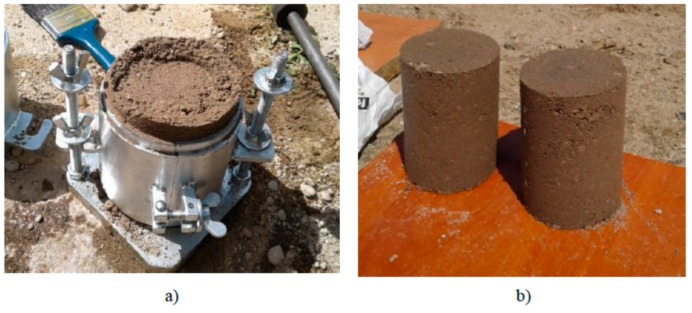
Preparation of the samples made of fine aggregate with cement: cylindric form (**a**); samples (**b**).

The mixture was poured into special cylindrical forms. Samples were prepared in special cylindrical forms of dimensions of Ø 100 mm; h = 115 mm (Figure 6a,b). The samples were compacted in three layers, 25 strokes per layer, by means of the Proctor method according to standard EN 13286-2, using a Ø 50 mm compactor of a weight of 2.5 kg, falling freely from a height of 305 mm. In total, 18 cylindrical samples of the dimensions of Ø 100 mm and h = 115 mm were made, for compressive strength testing at 7, 28, and 56 days. There were six samples tested on each date of the test. Additionally, six cubic samples of dimensions of 10 × 10 × 10 cm^3^ were made for frost resistance testing. 

Samples, having been prepared, were stored for 24 h in a mold, at a temperature of 20 ± 2 °C, followed by subsequent storage in a chamber, in the humidity of 95%–100% and temperature of 20 ± 2 °C and protected against drying. Three days before the test, the samples were submerged in water of the temperature of 20 ± 2 °C. One hour before compressive strength testing, the samples were taken out of the water and their surface was dried. Volume density, compressive strength, and frost resistance coefficient were tested.

The material with GEGA was proposed as a new solution. The mixture of the following composition was designed: CEM II/A-V 42.5 N, 205 kg; NATU, 90 kg; GEGA, 150 kg; water, 125 kg. The components of the mixture were mixed in a mechanical mixer. First, the slurry, composed of cement and water, was mixed for 2 min. Then, NATU was added and the mortar was mixed for next 1 min. GEGA was added to the mortar and mixed for 2 min.

**Figure 7:**
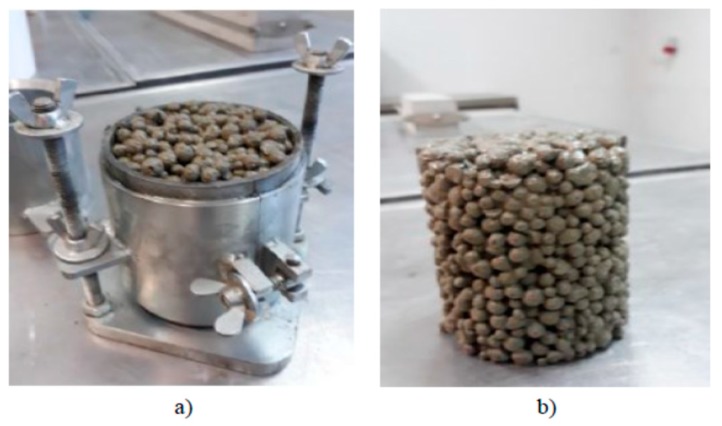
Preparation of a sample mixture with GEGA (**a**). Sample of the mixture with GEGA for compression strength tests (**b**).

The mixture was laid to special cylindrical molds of Ø 100 mm; h = 115 mm. The mix was compacted 25 times in two layers. The compaction method in accordance with the EN 206 standard is suggested by the authors. During concrete compacting, a hand-rammer of Ø 50 mm and weight of 1 kg was used. In order to ensure proper merger of the components, it is important to properly compact permeable ready mix with GEGA. It should be noted that too much compaction energy will reduce the porosity of the concrete, which, in turn, will reduce its permeability. Additionally, compacting shall be carried out in a way that prevents damage of the grains of the lightweight GEGA aggregate. For the compressive strength testing at 7, 28, and 56 days, 18 cylindrical samples of Ø 100 mm and h = 115 mm were made as shown in Figure 7a,b. There were six samples, tested on each assigned date of the tests. Additionally, 6 cubic samples of dimensions of 10 × 10 × 10 cm^3^ were made for frost resistance testing. Test samples were stored for 24 h in molds at a temperature of 20 ± 2 °C, followed by subsequent storage in a chamber at a humidity of 95%–100% and temperature of 20 ± 2 °C and protected against drying. One hour before the resistance test, the samples were taken out of the chamber and left to dry in the air at a temperature of 20 ± 2 °C. Volume density, compressive strength, and frost resistance coefficient were tested.

### 2.4. Test Methods

#### 2.4.1. Volume Density Test and Porosity Test

Volume density was determined for three samples, made of NATU and for three samples of the material, made of GEGA:

(1) $$\rho_{0}=\frac{m_{0}}{V},$$

where *ρ*_0_ is the volume density of the materials, *m*_0_ is the weight of the cylindrical sample, and *V* is the volume of cylindrical sample.

Volume density tests were carried out on cylindrical samples, intended for compressive strength testing. The volume of the sample was determined by measuring the sample’s dimensions.

Material porosity was marked as $f_{V}$ and defined as a ratio of the pores volume in the cylindrical sample to the total volume V of the sample. Porosity is the ratio of the volume of pass-through pores in a unit of the volume of a sample:

(2) $$f_{V}=\frac{V_{p}}{V},\;0<f_{V}\leq 1$$

Due to the relationship:

(3) $$V=V_{p}+V_{S}$$

Volume porosity is defined as:

(4) $$f_{V}=\frac{\left(V-V_{S}\right)}{V}=1-\frac{V_{S}}{V}$$

where $f_{V}$ is the material porosity, *V* is the total volume, *V_p_* is the volume of pores, and *V_s_* is the volume of the solid material.

A porosity test was carried out on three cylindrical samples. The sample volume was determined by the measurement of its dimensions. The volume of open pores was determined by measuring water volume which permeated inside the sample. Water, filling in the pores, was measured by capillary forces, without any external pressure. The pore volume *V_p_* was obtained by determining the mass of water that penetrated the pores of the sample. It has been assumed that 1 dm^3^ of water is equal to 1 kg.

#### 2.4.2. Compressive Strength Tests

The compressive strength test was carried out in the Advantest 9, Controls, Liscate MI, Italy) in a uniaxial state of stress in accordance with the procedure, defined in EN 13286-41. For the compressive strength testing at 7, 28, and 56 days, 18 cylindrical samples of Ø 100 mm and h = 115 were made. On each assigned date six cylindrical samples were tested. Compressive strength of the material was determined on the basis of the arithmetic mean of the results for all six. It was assumed that none of the results can deviate from the mean more than 10%. Standard deviation was determined.

#### 2.4.3. Frost Resistance Coefficient Tests

Samples for frost resistance coefficient test were stored for 28 days in a chamber with the humidity of 95%–100%, and temperature of 20 ± 2 °C. Then, they were submerged in water for 24 h, and placed in a frost chamber. Then they underwent 14 frost and thaw cycles. One cycle procedure involves freezing the samples for 8 h in the temperature of −23 ± 2 °C and thawing them for 16 h in the temperature of 18 ± 2 °C. Frost resistance coefficient was determined for three samples in accordance with Equation (5). The average compression strength *R_c_^Z−O^* was adopted for the calculations. The following frost resistance coefficient was adopted:

(5) $$M=\frac{R_{C}^{Z-O}}{R_{C}}$$

for *M_min_* = 0.7; where $R_{C}^{Z-O}$ is the average compressive strength of the samples, subjected to freeze-thaw cycles, and *R_c_* is the average compressive strength of reference samples (cared in air and water).

## 3. Results and Discussion

### 3.1. Volume Density Tests

**Figure 8:**
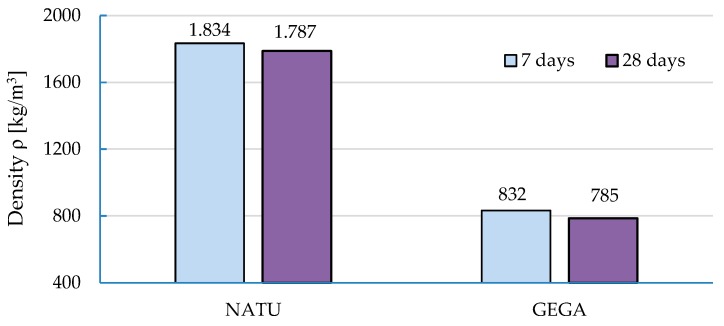
Change in apparent density.

The volume density of NATU and GEGA were determined at seven and 28 days, by measuring and weighing cylindrical samples according to Equation (1). The result is the arithmetic mean of the values, determined for six NATU and six GEGA cylindrical samples. The results are presented in Figure 8.

### 3.2. Compressive Strength Tests

**Figure 9:**
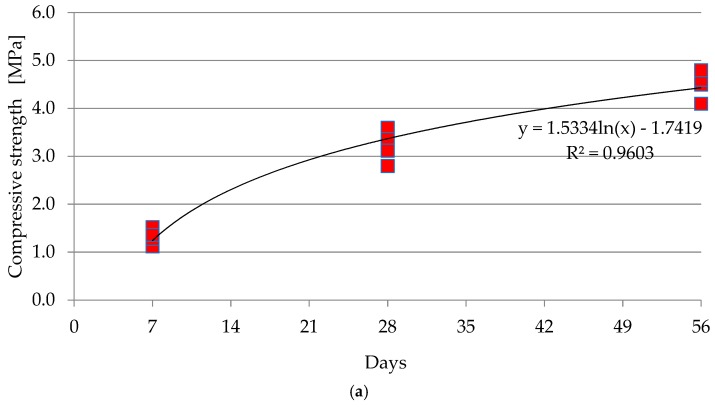

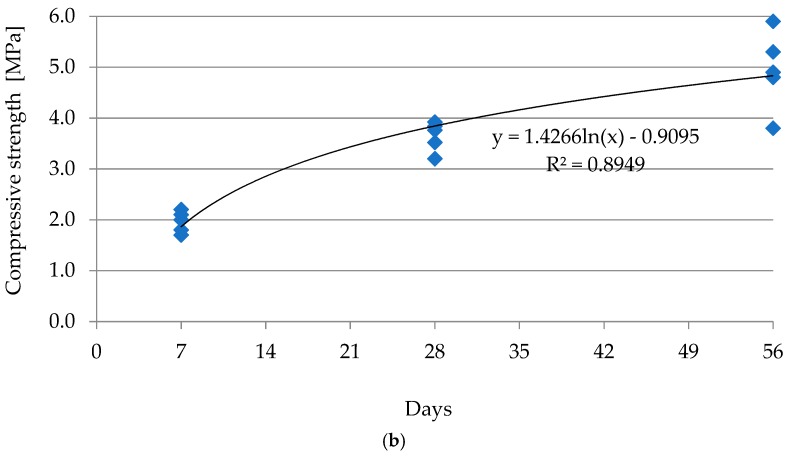
Growth of compression strength with time: (**a**) NATU, (**b**) GEGA.

The average compressive strength of NATU, tested on six samples, was 1.3 MPa (standard deviation s = 0.2 MPa), while the average compressive strength of the six samples (cylinders) on the 28th day was 3.2 MPa (s = 0.4 MPa). At 56 days, the average compressive strength was 3.8 MPa (s = 0.3 MPa). It is shown in Figure 9a. Compressive strength test of the material with GEGA was performed at seven days on six cylindrical samples and it was 1.9 MPa (s = 0.2 MPa), while on the 28th day the average compressive strength of the six cylindrical samples was 3.7 MPa (s = 0.3 MPa). At 56 days, the average compressive strength was 4.9 (s = 0.7 MPa). Test results are presented in Figure 9b.

The carried out frost resistance tests of NATU and GEGA have shown that both materials have met Polish Technical Requirements WT-5 2010, “Mixtures bound with a hydraulic binder for national roads” and that the frost resistance index, calculated according to Equation (5), was higher than the required minimum M_min_ = 0.7, where the NATU frost resistance index was 0.74 and the GEGA frost resistance index was 0.94.

## 4. Analysis and Application of the Traditional Pavement Structure and with Use of a GEGA Permeable Layer

Taking into account favorable properties of permeable concrete, the analysis of the possibilities to apply this material for a road course in typical solutions for pavement structure was carried out in accordance with Polish requirements [45,46].

The procedure for the calculation of the required thickness of the lightweight GEGA permeable concrete course, as an alternative to a typical frost protection course and improved soil sub-grade, was carried out in accordance with the guidelines, contained in [47,48,49]. For each of the analyzed solutions the values of elastic deflections “w” on the surface of the improved sub-grade course and on the surface of the sub-base courses of the pavement structure were determined. Calculations were carried out using BISAR 3.0 software (Bitumen Stress Analysis in Roads, Shell, Gdansk, Poland). The required *E_equivalent_* substitute modulus on the surface of the analyzed layers was calculated by means of using the Boussinesq equation (Equation (6)) from the theory of elastic half-space, which is the following:

(6) $$E_{equivalent}=\frac{q\times D\times (1-v^{2})}{w},$$

where *E_equivalent_* is the substitute modulus, determined on the surface of sub-base course layer of pavement structure and on the surface of improved subgrade, MPa; *q* is the contact pressure of the wheel *q* = 0.65 kPa; *D* is the substitute diameter of the wheel tract, *D* = 0.313 m; *ν* is Poisson’s ratio, *ν* = 0.3; and *w* is the deflection on the surface of road profile, m.

Table 4, Table 5 and Table 6 summarize the proposal of application of lightweight concrete sub-grade course containing GEGA material instead of traditional frost-resistant layer and improved sub-grade course. Calculations of the required thickness of the GEGA concrete road pavement layer for flexible and rigid pavements were carried out, depending on the traffic category, i.e., the traffic load. The results of the calculations, presented in Table 4, Table 5 and Table 6, refer to three selected representative road traffic categories, according to [44], representing heavy, medium, and light traffic.

The computational analysis showed that it is possible to replace the traditional solution of the frost protection course and the improved soil sub-grade course with a single course of lightweight concrete containing GEGA material. The proposed alternative solution which may be used in both flexible and rigid pavement structures, features the required load-bearing capacity and durability parameters.

## 5. Conclusions

The completed tests and analyses allow the following conclusions to be drawn:

1. The research confirmed that the road sub-grade of permeable concrete, made of GEGA, has similar mechanical properties to a sub-grade with NATU and can function as a frost protection layer for the road sub-base. Additionally, the material with GEGA is permeable to liquids. Therefore, it is possible to obtain a lightweight material with GEGA of the mechanical properties, meeting the requirements, imposed on the frost protection layer, made of natural aggregate NATU, stabilized with a hydraulic binder. Thus, it is possible to substitute the sub-grade and the frost-protection sub-base with a single layer, made of lightweight material with GEGA.
2. The analysis has shown that practical implementation of the proposed solution is technically justified in the case of flexible and rigid pavements. Application of the recycled material with GEGA allows an effective construction of the road pavement, maintaining the required load-bearing capacity and durability parameters.
3. The proposed solution, concerning the use of recycled materials is fully coherent with the idea of environment protection and sustainable development of the economy, not only in Europe, but worldwide. The application of GEGA allows limiting the mining of the NATU materials, due to which environmental degradation can be reduced.
4. It is recommended to carry out further research on the LCC (life cycle costs) analysis, conducted for flexible and rigid pavements, for selected traffic categories and for two technologies of road pavement construction [50,51,52].
5. Further research into lightweight aggregate’s compressive strength increase shall be carried out and the elastic modulus shall be determined, as well as volume flow rate, depending on the porosity.
6. Rapid development in industrial application of GEGA and other lightweight aggregates, manufactured from recycled materials, requires a proposal and implementation of the design methods for the materials with synthetic aggregates and recycled aggregates.

## Figures and Tables

**Table 1:** Chemical content and physical properties of the cement CEM II/A-V 42.5 N.

Setting Start Time (min)	Setting End Time (min)	Compressive Strength (MPa)			Blaine Fineness (cm^2^/g)	Coal Content (%)	Water Demand (%)
		2 days	28 days				
**195**	**265**	**21.3**	**51.7**		3521	3.0	28.5
**Content (%)**							
SiO_2_	Al_2_O_3_	Fe_2_O_3_	CaO	MgO	SO_3_	Na_2_eq	Cl
25.0	8.8	3.2	54.0	1.2	2.6	0.86	0.042

**Table 2:** Chemical composition of the aggregate.

Aggregate Type	Content (%)									
	SiO_2_	Al_2_O_3_	Fe_2_O_3_	CaO	MgO	SO_3_	Na_2_O	K_2_O	TiO_2_	Coal Content
NATU	97.5	0.8	0.08	0.3	-	0.05	-	-	0.1	-
GEGA	63.3	0.74	-	14.19	2.98	0.32	13.35	0.57	-	4.53

**Table 3:** Physical properties of the aggregates.

Property		NATU	GEGA
Water absorption WA_24_	(%)	1.8	17.8
Particle density ρ_a_	(kg/m^3^)	2640	350
Oven dried particle density ρ_rd_	(kg/m^3^)	2520	310
Saturated surface-dry particle density ρ_ssd_	(kg/m^3^)	2560	330
Porosity P	(%)	n.s.	42
Crumble indicator *X_r_*	(%)	n.s.	25.9
pH after 24 h	-	n.s.	11.9
Loose bulk density *ρ_b_*	(kg/m^3^)	1550	180
Compact bulk density *ρ_c_*	(kg/m^3^)	1760	255
Thermal conductivity of 40 cm layer of the aggregate	W/m·K	n.s.	0.69

n.s.—not studied.

**Table 4:** Resulting structural layers of flexible and rigid pavements—traditional and permeable concrete—made of GEGA, solutions for heavy traffic.

Type of Pavement							
Traffic Load—Heavy Traffic							
22.00–52.00 mln of ESAL 100 kN/lane in the design period of 30 years				42.63–101.25 mln of ESAL 100 kN/lane in the design period of 30 years			
FLEXIBLE PAVEMENT with asphalt mixture layers and base course, made of unbound mixture TRADITIONAL SOLUTION		FLEXIBLE PAVEMENT with asphalt mixture layers and base course, made of unbound mixture GEGA SOLUTION		RIGID PAVEMENT with concrete slab in wearing layer and base course, made of mixture, bound with hydraulic binder TRADITIONAL SOLUTION		RIGID PAVEMENT with concrete slab in wearing layer and base course, made of mixture, bound with hydraulic binder GEGA SOLUTION	
4 cm	Wearing course, made of asphalt mixture	4 cm	Wearing course, made of asphalt mixture	27 cm	Wearing course, made of cement concrete—dowelled and anchored	27 cm	Wearing course, made of cement concrete—dowelled and anchored
8 cm	Binder course layer, made of asphalt concrete	8 cm	Binder course layer, made of asphalt concrete	-	Slip layer: surface dressing or geotextile fabrics	-	Slip layer: surface dressing or geotextile fabrics
16 cm	Base course layer, made of asphalt concrete	16 cm	Base course layer, made of asphalt concrete	18 cm	Base course layer, made of mixture bound with 8/10 hydraulic binder	18 cm	Base course layer, made of mixture bound with 8/10 hydraulic binder
20 cm	Base course layer, made of unbound mixture with C 90/3 aggregate	20 cm	Base course layer, made of unbound mixture with C 90/3 aggregate	15 cm	Sub-base layer, made of mixture, bound with C5/6 hydraulic binder	15 cm	Sub-base layer, made of mixture, bound with C5/6 hydraulic binder
15 cm	Sub-base layer, made of mixture, bound with hydraulic binder	15 cm	Sub-base layer, made of mixture, bound with hydraulic binder	20 cm	Frost resistant layer, made of unbound mixture with a function of drainage layer	**28 cm**	**Permeable concrete, made of GEGA**
20 cm	Frost resistant layer made of unbound mixture, with a function of drainage layer	**28 cm**	**Permeable concrete, made of GEGA**	25 cm	Improved sub-grade, made of soil, stabilized with hydraulic binder		
25 cm	Improved sub-grade, made of soil stabilized with hydraulic binder			Subgrade, native soil—excavation			
Subgrade, native soil—excavation							

**Table 5:** Resulted structural layers of flexible and rigid pavements—traditional and permeable concrete, made of GEGA, solutions for medium traffic.

Type of Pavement							
Traffic Load—Medium Traffic							
2.50–7.30 mln of ESAL 100 kN/lane in the design period of 30 years				6.39–15.99 mln of ESAL 100 kN/lane in the design period of 30 years			
FLEXIBLE PAVEMENT TRADITIONAL SOLUTION		FLEXIBLE PAVEMENT GEGA SOLUTION		RIGID PAVEMENT TRADITIONAL SOLUTION		RIGID PAVEMENT GEGA SOLUTION	
4 cm	Wearing course, made of asphalt mixture	4 cm	Wearing course, made of asphalt mixture	23 cm	Wearing course, made of cement concrete—dowelled and anchored	23 cm	Wearing course, made of cement concrete—dowelled and anchored
6 cm	Binder course layer, made of asphalt concrete	6 cm	Binder course layer, made of asphalt concrete	-	Slip layer: surface dressing or geotextile fabrics	-	Slip layer: surface dressing or geotextile fabrics
10 cm	Base course layer, made of asphalt concrete	10 cm	Base course layer, made of asphalt concrete	20 cm	Base course layer, made of mixture, bound with C 5/6 hydraulic binder	20 cm	Base course layer, made of mixture, bound with C 5/6 hydraulic binder
20 cm	Base course layer, made of unbound mixture with C 90/3 aggregate	20 cm	Base course layer, made of unbound mixture with C 90/3 aggregate	15 cm	Sub-base layer, made of mixture, bound with C 3/4 hydraulic binder	15 cm	Sub-base layer, made of mixture, bound with C 3/4 hydraulic binder
15 cm	Sub-base layer, made of mixture, bound with hydraulic binder	15 cm	Sub-base layer, made of mixture, bound with hydraulic binder	20 cm	Frost protection layer, made of unbound mixture; with a function of drainage layer	**23 cm**	**Permeable concrete, made of GEGA**
20 cm	Frost protection layer, made of unbound mixture, with a function of drainage layer	**23 cm**	**Permeable concrete, made of GEGA**	25 cm	Improved sub-grade made of soil, stabilized with hydraulic binder		
25 cm	Improved sub-grade, made of soil, stabilized with hydraulic binder			Subgrade, native soil—excavation			
Subgrade, native soil—excavation							

**Table 6:** Resulted structural layers of flexible and rigid pavements—traditional and permeable concrete, made of GEGA, solutions for light traffic.

Type of Pavement							
Traffic Load—Light Traffic							
0.09–0.50 mln of ESAL 100 kN/lane in the design period of 30 years				0.15–0.75 mln of ESAL 100 kN/lane in the design period of 30 years			
FLEXIBLE PAVEMENT TRADITIONAL SOLUTION		FLEXIBLE PAVEMENT GEGA SOLUTION		RIGID PAVEMENT TRADITIONAL SOLUTION		RIGID PAVEMENT GEGA SOLUTION	
4 cm	Wearing course, made of asphalt mixture	4 cm	Wearing course, made of asphalt mixture	24 cm	Wearing course layer, made of cement concrete—undowelled	24 cm	Wearing course layer, made of cement concrete—undowelled
8 cm	Binder course layer, made of asphalt concrete	8 cm	Binder course layer, made of asphalt concrete	30 cm	Base course layer, made of unbound mixture, made of C 50/30 aggregate	30 cm	Base course layer, made of unbound mixture, made of C 50/30 aggregate
20 cm	Base course layer, made of unbound mixture with C 90/3 aggregate	20 cm	Base course layer, made of unbound mixture with C 90/3 aggregate	22 cm	Frost protection layer made of unbound mixture; with a function of drainage layer	**18 cm**	**Permeable concrete, made of GEGA**
22 cm	Frost protection layer, made of unbound mixture; with a function of drainage layer	**18 cm**	**Permeable concrete, made of GEGA**	24 cm	Improved sub-grade, made of soil, stabilized with hydraulic binder		
24 cm	Improved sub-grade, made of soil, stabilized with hydraulic binder			Subgrade, native soil—excavation			
Subgrade, native soil—excavation							
